# What makes teaching joyful? Unpacking the wellbeing-resilience-enjoyment chain in higher education faculty

**DOI:** 10.3389/fpsyg.2025.1708425

**Published:** 2026-01-06

**Authors:** Zixiang Zhao, Dan Yang, Po-Wen Tsai

**Affiliations:** International Business School, Jilin International Studies University, Changchun, China

**Keywords:** emotional resilience, enjoyment, institutional support, self-efficacy, teaching

## Abstract

**Introduction:**

This study investigates the psychological and organizational determinants of enjoyment in teaching by university instructors. The study hypothesizes a model in which teacher self-efficacy and well-being enhance enjoyment in teaching through emotional resilience as a mediator and institutional support and professional commitment as moderators.

**Methods:**

This study employed a cross-sectional survey design with purposive sampling of 273 faculty members from Chinese universities, using validated Likert-scale instruments. Data were analysed using SPSS and AMOS through correlation, regression, and structural equation modelling to test mediation and moderation effects, alongside reliability and validity assessments.

**Results:**

The findings indicated that well-being and self-efficacy were strong predictors of teaching enjoyment. Emotional resilience was found to mediate those relations, translating individual strengths into long-term joy of teaching. While professional commitment was a significant moderator of the emotional resilience-teaching pleasure relationship, institutional support had no moderating influence. The findings detail the central role of inner psychological abilities and professional identity in enriching beneficial teaching experiences.

**Discussion:**

The study adds to the teacher well-being literature base and provides an enhanced understanding of the part that resilience can play in mediating internal assets and job satisfaction. The study offers valuable insights towards the development of emotionally healthy learning spaces and enhancing faculty engagement using a strength-based approach. This perspective aligns with a strength-based approach. It emphasizes building internal psychological resources such as self-efficacy, well-being, and resilience rather than focusing on deficits or problems.

## 1 Introduction

These days, more attention is being given to the feelings and well-being of faculty members in higher education, particularly how they manage and sustain their sense of joy in their jobs. It was once thought that teaching is primarily about thinking and instructing; however, recent studies reveal that educators also invest considerable emotional and psychological effort in their jobs, as discussed by Kamboj and Garg (2021). Feelings of joy, satisfaction, resilience, happiness, and commitment of the faculty, their length of employment, and how involved they are with the institution strongly influence good teaching. Given the current situation, knowing what makes teaching joyful is very important and necessary.

Enjoyment in teaching is different from overall satisfaction and describes the intimate joy and involvement teachers have when they teach (Thumvichit, 2024). Emotional resilience helps faculty cope with challenges, and support from institutions and professional commitment which strengthen their ability to sustain joy in teaching (Brashovetska, 2023). Enjoying what teachers do in school has been shown to make students more motivated, more creative, and eager to participate. Self-efficacy refers to the perceived capabilities of doing tasks well (Schunk and DiBenedetto, 2021), and it is an important factor leading to teachers enjoying their work. Self-efficacy, well-being, and emotional resilience contribute to teachers’ ability to enjoy and cope with their work.

Emotional resilience is also greatly influenced by the general well-being of staff, a concept involving emotional, psychological, and social aspects. People who have a better sense of well-being among teachers tend to handle their jobs more calmly (Nurfatihah Razali and Sukor, 2023), are less prone to burnout, and deal with stress more effectively. If faculty members enjoy support and autonomy, as explained in self-determination theory, they usually show greater happiness and resilience in their teaching. The ability to cope with stress is what links our self-esteem, wellness, and general satisfaction at work to how much we like teaching. Well-being and institutional support also shape faculty enjoyment and resilience. Support within the institution, such as leaders who pay attention, give access to resources, and show appreciation, can greatly uplift faculty mood and teaching motivation (Zhao, 2024). Similarly, professional commitment is an indication of how much teachers relate to teaching, appreciate its purpose, and are internally driven to help students. Those who are professionally committed tend to handle problems, improve the way they teach, and feel happy with their work. Nobody has yet constructed a unifying framework for examining how self-efficacy and well-being might affect teachers’ happiness by building emotional resilience, and how this happens when affected by workplace and professional factors in colleges and universities.

Existing research links teacher self-efficacy to better well-being and performance, but important gaps undermine a coherent causal model for higher education faculty. Most studies foreground primary and secondary educational contexts or general well-being without isolating teaching enjoyment as a distinct outcome for university teachers, leaving faculty-specific mechanisms underexamined (Wang et al., 2024a; van Dijk et al., 2024). Emotional resilience is often discussed as an individual resource, yet it is rarely tested as the mediating pathway that transforms self-efficacy into sustained enjoyment of teaching, producing an empirical blind spot about how adaptive emotion regulation operates between beliefs and positive affect in teaching (Bagdžiūnienė et al., 2023). Institutional support and professional commitment are each linked to well-being, but their joint moderating roles (how organizational resources and vocational identity amplify or buffer the resilience-enjoyment link) remain underpowered or untested in recent studies (Bastian and Widodo, 2024). Besides, there is a scarcity of faculty-focused context-sensitive investigations that capture post-pandemic shifts in workload, hybrid teaching, and digital burnout, which plausibly changed the dynamics of efficacy, resilience, and enjoyment (Yang and Du, 2024; Emiru and Gedifew, 2024). Thus, these gaps justify a targeted, theory-driven test of the proposed model now, as higher education is undergoing rapid pedagogical and occupational change, so understanding the mechanisms and boundary conditions that foster joyful teaching is urgent for retention, quality, and faculty well-being.

Situating the research in a university setting helps address the gap in current literature and adds more insights into what faculty members go through. It helps the academic community by addressing the issue of faculty happiness, not just stress, and exploring how happiness is sustained in university teaching. Hence, the purpose of the study is to develop and use a model of the chain between well-being, resilience, and enjoyment among higher education faculty. This study adopts a strength-based approach, which emphasizes positive internal capacities of teachers such as confidence, emotional stability, and well-being. These are the key drivers of resilience and joyful teaching. The objective of the study is to investigate the relationship between teacher self-efficacy, well-being, and teaching enjoyment. Another objective is to study whether emotional resilience acts as a bridge between internal resources (self-efficacy, well-being) and teachers’ enjoyment of their work. Lastly, to measure the moderating impact of institutional support and professional commitment on emotional resilience and teaching enjoyment. By handling these objectives, the study helps build a strong literature on faculty mental health, teachers’ readiness for problems, and engagement with teaching in higher education. As a result, HR departments, university executives, and those responsible for training have practical tools.

## 2 Literature review

### 2.1 Enjoyment of teaching in higher education

Teaching enjoyment is the pleasant emotional experiences and feeling of happiness when teaching, as well as while interacting with students (Burić and Wang, 2024). While job satisfaction covers a broader set of workplace experiences, salary, and tasks, teaching enjoyment is mainly about the inner joy and gratification found in lesson planning, working with students, and watching them learn. Faculty who enjoys their work tend to teach in student-centered ways, think of new learning activities, and encourage students to learn more through creative methods. In addition, this enjoyment strengthens teachers’ motivation, support for their career choice, and lasting job satisfaction. According to Self-Efficacy Theory, individuals judge their capabilities to organize and execute certain actions (Bhati and Sethy, 2022).

But so far, studying teaching enjoyment in higher education has not been common. Most studies in this field study teachers or highlight issues like burnout and emotional exhaustion. The researcher wants to change the conversation to focus on what supports joy and positive feelings in university teaching instead of identifying what teachers lack. New perspectives from positive psychology recommend encouraging joy in teachers because it helps protect them from stress and builds their resilience (Wang et al., 2021). In line with a strength-based perspective, positive psychology highlights the importance of reinforcing internal strengths of teachers, such as self-efficacy, optimism, and emotional resilience. It can promote greater enjoyment and engagement, rather than focusing on deficits or burnout. In brief, thinking about teaching enjoyment as a special construct plays an important role in grasping faculty engagement and performance. It indicates a person’s mental well-being as well as how healthy the overall school setting is. Looking for positive aspects of teaching, schools, and educators can figure out how to help faculty feel better, teach more effectively, and improve student results.

### 2.2 Teacher well-being and its role in engagement

Happiness is very important for a productive workplace, longevity, and general enjoyment of work (Kartilah et al., 2023). Basically, it refers to an active state covering psychological, emotional, and social wellness, which allows teachers to excel in their profession as well as their personal lives. At universities, being healthy for faculty includes feelings of purpose, being accepted, making autonomous decisions, and being recognized (Hammoudi Halat et al., 2023). Self-determination theory helps in addressing individual motivation and well-being (Ryan et al., 2022). The Self-Determination Theory (SDT) makes it easier to understand how educators feel satisfied by highlighting the need for autonomy, competence, and relationships. Teachers who plan their syllabus, guide students well, and have positive relationships at work are more likely to be content. Many studies have shown that having a sense of fulfillment in teaching leads to better teaching involvement, less teacher burnout, and longer motivation (Wang et al., 2024b). A culturally adapted mindfulness program helped pre-service teachers improve their mindfulness, self-compassion, and life satisfaction, according to Wu and Qin (2025). They underline the role culturally responsive approaches play in teacher education to help maintain emotional and professional health, success, and adaptation in a wide range of schools.

The problems affecting teacher well-being in higher education are frequently related to structure and feelings. Teachers in academia regularly handle large workloads, many administrative tasks, and unclear job roles, and do not always get sufficient recognition for the teaching they do. Experiencing too much stress at work can reduce satisfaction and harm workers’ emotional well-being (Segal et al., 2025). On the other hand, when institutions offer guidance, resources, and psychological support to their staff, faculty well-being gets better, which encourages them to keep engaging in their teaching work. According to Roos and Borkoski (2021), faculty well-being is important for the growth of academia. It has also been found that well-being results in students being more flexible in the classroom and teachers experimenting more in their approach.

Emotional resilience, shaped in part by well-being, helps individuals cope with future events at work (Kumar Pradhan et al., 2021). Those with well-being profiles can display positive psychological qualities like optimism and emotional stability, allowing them to keep enjoyment alive for students during stressful teaching situations. So, having a good level of well-being may not only come from the right support, but it can also lead to a better teaching experience and improved resilience. Teaching satisfaction and mental toughness of higher education teachers start with their happiness and well-being. It keeps faculty in a positive state, maintains their enthusiasm, and enables them to teach excitingly. Universities that pay attention to faculty well-being tend to develop teachers who are stronger, happier, and more supportive, which is an added advantage for students, too.

### 2.3 Self-efficacy and teaching motivation

An educator’s belief in their ability to take certain actions to achieve what is needed shows up as both motivation and success in their roles. In the field of teaching, self-efficacy is about a teacher’s confidence in organizing lessons, managing classroom difficulties, providing good instruction, and affecting students’ learning (Shah, 2023). Their beliefs about teaching play an important role in how teachers do their job, handle challenges, and feel about their achievements. Teachers in higher education must handle topics in their field, class diversity, evaluation, and maintaining class involvement. Having this kind of resilience, creativity, and commitment to their job leads teachers to experience better outcomes for themselves and their students. Significantly, such teachers are much more intrinsically motivated because they believe they can make a difference in education. According to Linge et al. (2021), there are four different ways someone might gain self-efficacy: through mastering the skill personally, observing someone succeed, hearing encouragement, and through their emotions and bodily sensations. Having positive experiences teaching in the past is found to be the strongest influence on a person’s self-confidence. Regularly succeeding in guiding different students between classes gains the faculty’s confidence and motivates them to try new teaching methods. Having failures, unfavorable experiences, or poor reviews can bring down a person’s belief in their abilities and desire to be innovative (Ng et al., 2022).

Research points out the favorable effect of self-efficacy on job satisfaction, feelings of well-being, and involvement in class. It additionally protects people from stress and burnout by encouraging them to handle challenges and keep their motivation (Li, 2023). Instructors who believe in their skills usually have a better time teaching since they believe they can address challenges and make a difference in students’ achievements. Also, having a sense of efficacy helps people build emotional resilience. When educators are sure of themselves, they are better at controlling their emotions, adjusting to change, and staying positive through pressures at school or at work. Because of this resilience, teachers can enjoy their work more, knowing their strong emotional foundation will support positive lessons. As a result, self-efficacy helps teachers feel joy in their jobs by influencing their mental toughness and motivation to keep going. All in all, self-efficacy is a key psychological skill needed in higher education, as it is associated with increased teaching quality and teaching practices (Na and Isa, 2024). It gives faculty the skills to manage difficulties in instruction, maintain motivation, and find satisfaction in their responsibilities. Therefore, strengthening self-efficacy with professional programs, peer mentoring, and feedback should be a priority for happy and resilient teachers today.

### 2.4 Emotional resilience as a mediating resource

An emotionally resilient person can face adversity, stress, and uncertainty while staying stable emotionally and mentally (Sisto et al., 2019). Because faculty members in higher education deal with many different demands, such as changing schedules, student issues, reforms at the institution, and pressure to perform well, being emotionally resilient is necessary for both their well-being and job involvement. Having positive emotions, keeping motivated, and raising your professional skills are just as important as getting through any setbacks. Those faculty members who feel confident and have good well-being tend to work on their emotional control, look at situations from different angles, and adjust their actions, which helps them be more resilient. Due to resilience, they can react optimistically to challenges, keeping their motivation up as teachers. Bagdžiūnienė et al. (2023) concluded that resources on the job and confidence in success play a major role in developing emotional resilience among teachers, which then benefits their well-being. Resilience affects the way resources and well-being are linked, but not how much someone might consider leaving their job. They stress that resilience plays an important role in helping educators keep their emotions stable.

When teachers have high resilience, emotional exhaustion is eased, and they enjoy teaching more. Likewise, Taylor (2013), pointed out that resilience gives educators the ability to use what they have inside and outside their work to face issues at school and maintain their passion. Teachers who show resilience make their own decisions more confidently, tend to focus on resolving problems, and are committed to teaching over the long run (Tait, 2008). At higher education institutions, where professors must balance research, teaching, and service, resilience allows them to prioritize, look at situations differently, and keep enjoying teaching. Resilient faculty members can bounce back from setbacks in teaching, make use of feedback positively, and excite students with great spirit. This adaptability in emotions supports a long-lasting joy in teaching and helps them avoid being burnt out. Professional training, teamwork, and a reflective process usually help teachers develop resilience by interpreting challenges and gaining knowledge from what they go through. They help this by setting up supportive spaces, giving recognition to teachers, and supplying mental resources for coping and adjusting. In short, emotional resilience acts as an important connector between qualities like happiness, trusting in one’s abilities, and enjoying teaching success. Resilience strengthens adaptive emotions and ways of dealing with challenges and it changes psychological readiness into lessons that teachers enjoy, making it an essential link in the well-being–resilience–enjoyment chain.

### 2.5 Organizational environment and faculty well-being

The term institutional support means how organizations and leaders help members succeed both in their work and perform in a productive manner (Falola et al., 2020). Help for faculty in higher education might cover teaching materials, administrative guidance, mentorship, helpful comments, and acknowledgement of teaching involvement. An encouraging environment at an institution helps faculty members deal with how emotionally draining teaching can be and encourages them to stay interested in their jobs. Studies demonstrate that having strong institutional backing helps faculty members feel better and cope better with stress. Those who work in schools and the education field and feel important and empowered by their department and leaders are more likely to be satisfied with their job, stay motivated, and remain committed for the long term. On the other hand, not getting support from colleagues can lead to a sense of isolation, make it more difficult for people to develop professionally, and increase emotional stress, all of which can make teaching less enjoyable. The study conducted by Gast et al. (2022), indicates that professional development workshops, reflection, and involvement in learning communities can support new faculty in achieving better well-being, especially through more self-confidence, growth, and a sense of purpose at work. The evidence suggests that using targeted learning methods can strengthen and maintain early-career faculty’s emotional resilience.

Support provided by organizations strengthens emotional toughness by cutting down unnecessary stress and giving a feeling of safety. According to Grensing-Pophal and Steven (2022), managers can respond to employees’ needs regarding mental health and help in maintaining work-life balance. Having systems that support faculty in trying out new teaching styles, managing students’ needs, and gaining access to helpful resources helps them cope with challenges. These situations help make resilience strong within people’s personal lives and also keep social support close when they need it at work. Also, being in encouraging surroundings helps individuals look at themselves and develop. Taking part in professional development, joining communities of practice, and using teaching innovation grants help faculty improve their skills and confirm their value as educators. Including these institutional aspects makes teaching a more trusted, independent, and meaningful experience, all important for teachers to stay happy in their profession. Hence, giving faculty support from institutions is not just optional; it greatly impacts how resilient and happy faculty members are. The emotional factors at an institution can support or interfere with the personal resources that shape engaging and pleasurable teaching. A strong institutional culture can greatly impact the well-being, resilience, and enjoyment chain among faculty in higher education.

### 2.6 Professional commitment and identity

An employee who demonstrates professional commitment is emotionally involved in the profession, identifies with it, and wants to keep the role. Within higher education, professors who show strong professional commitment see teaching as more than a job; they view it as personally important and as having significance for society. Having such a strong commitment helps educators handle tough times in education and stay dedicated to helping students and improving as teachers. According to Begum (2022), professional commitment refers to the loyalty and dedication of a person to stay in a profession. Professional commitment has been linked to being more persistent, happier on the job, and more resilient. Those who are enthusiastic about their jobs tend to spend more effort improving teaching, trying out new reflective practices, and staying active in their roles despite any pressures from the institution. According to Shao (2023), enthusiastic teachers tend to have fulfilling jobs. Because their professional identity is strong, counselors find it easier to cope emotionally and make sense of things in times of change at work. How committed teachers are affects the way emotional resilience is connected to how much they enjoy teaching. Strongly identified faculty members tend to view hurdles in teaching as an opportunity to learn and develop, instead of something that burdens them. Being motivated and guided by their values allows them to stay resilient and keep enjoying their jobs. In this case, professors who commit less to their profession may face emotional exhaustion, become less engaged, or become more cynical if they experience challenges. Additionally, a commitment to the profession usually grows because of personal considerations as well as feedback from others. Teachers feel more connected and valued by their institutions when institutions respect their values and support professional growth (Kainde and Mandagi, 2023). Feeling a sense of unity reinforces your strength and ability to enjoy teaching. On the whole, a strong commitment to work anchors teachers’ emotions and makes teaching more enjoyable. Workload supports educators to stay persistent, adjust to challenges, and make their work meaningful, which is important in the well-being, resilience, and enjoyment framework in universities.

### 2.7 Self-efficacy, well-being, and resilience

Faculty members’ belief in their capability to plan, enact, and adapt teaching activities (self-efficacy) provides the foundational personal resource that initiates the well-being-resilience-enjoyment chain. Increased self-efficacy correlates with better psychological well-being, such as positive working emotions and reduced burnout vulnerability (Wang et al., 2024a; Li, 2023). This allows a faculty member to experience a greater level of fulfillment and emotional wellness (the psychological resource) when the faculty member feels competent and autonomous. Good well-being, in turn, promotes the growth of emotional resilience. Self-efficacy and well-being are closely associated with resilience among teaching professionals (Lu et al., 2024; Salvo-Garrido et al., 2025; Zhang, 2023). This implies that faculty can be resilient to positive affective states even when faced with pressure. The connection between self-efficacy (and well-being) and outcomes as teaching enjoyment, is mediated by resilience, since well-being and beliefs are translated into long-term enjoyment of teaching. This conceptual integration follows positive-psychology orientations in higher education: teaching enjoyment is more likely to happen when internal resources are strong (efficacy, wellness), and when there is a dynamic interaction between them (resilience), leading to increased engagement and positive teaching behaviors.

### 2.8 The present study

There is now more attention on teachers’ happiness and emotional well-being in higher education; this study looks into what psychological and organizational factors affect teachers’ enjoyment of teaching. This research introduces a model focused on teacher self-efficacy and well-being influencing teachers’ enjoyment of work, as well as the roles of emotional resilience, institutions’ support, and teachers’ professional commitment. The purpose is to look at all the aspects that make teaching joyful, especially as it happens in the more emotional environment of higher education. The following hypothesis can be derived from the model:

*H1*: Teacher self-efficacy positively affects teaching enjoyment.

Believing that you can successfully perform tasks is called self-efficacy (Hussain et al., 2022). Depending on their self-efficacy, students might be more courageous, motivated, and emotionally collected, which leads to finding more joy in teaching. Consistent studies find that having high self-efficacy leads to more happiness in one’s job, stronger motivation, and greater satisfaction (Dös, 2023).

*H2*: Teacher well-being positively affects teaching enjoyment.

According to Van Dierendonck and Lam (2023), psychological well-being has six dimensions, including self-acceptance, positive relations with others, autonomy, environmental mastery, personal growth, and purpose in life. It helps people feel fulfilled emotionally and have a clear direction at work. Teachers who have good psychological health are more likely to enjoy and get a sense of purpose from teaching. Having good well-being supports positive emotions and motivates enthusiastic teaching (Shao, 2023).

*H3*: Emotional resilience mediates between teacher self-efficacy and teaching enjoyment.

The hypothesis states that emotionally resilient teachers use their confidence to make teaching both rewarding and worthwhile. People who have a strong belief in their abilities usually build good emotional skills, which help them continue feeling joyful in tense situations. So, resilience forms an important link between doing well and feeling good.

*H4*: Emotional resilience mediates the relationship between teacher well-being and teaching enjoyment.

Strong well-being builds emotional strength that allows teachers to maintain joy in their work, and it also correlates with student’s well-being (Kun and Gadanecz, 2022). Their emotions and job performance are beneficial when teachers are strong emotionally. Resilience can transfer a healthy mindset into more satisfaction at work and involve employees more deeply in their responsibilities.

*H5*: Institutional support moderates the relationship between emotional resilience and teaching enjoyment.

It is predicted that support from the institution will lead to resilience having a bigger impact on teaching enjoyment (Zhang, 2023). When teachers get emotional backing and develop professionally and a sense of community, their resilience results in better teaching practices.

*H6*: Professional commitment moderates the relationship between emotional resilience and teaching enjoyment.

Professional commitment impacts the way emotional resilience relates to a positive sense of teaching enjoyment. Even with strong emotional resilience, a teacher’s job satisfaction can change based on their passion for the job. Teachers who have a strong connection to their work are often able to enjoy their teaching.

**Figure 1:**
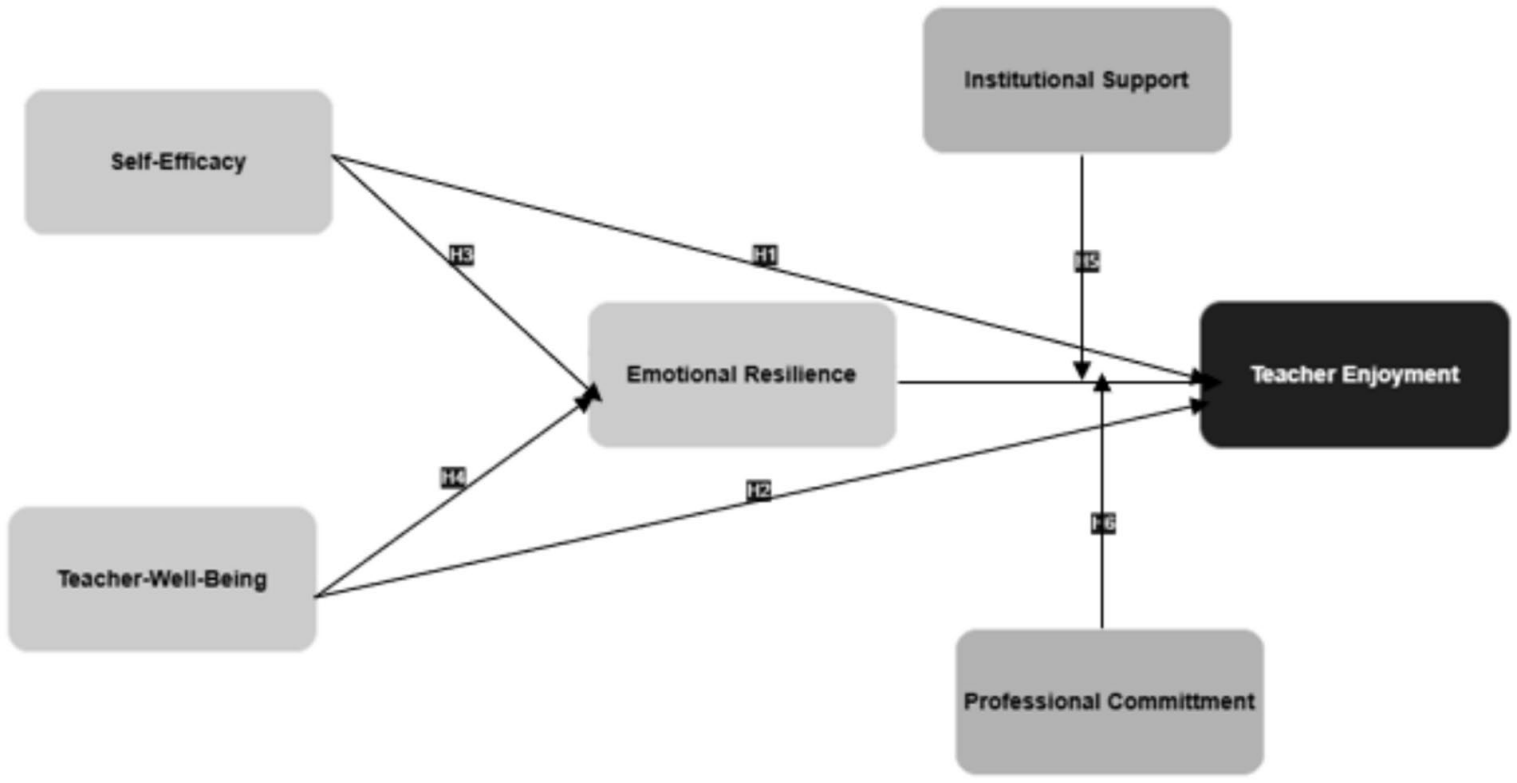
Conceptual model. Source: Author-generated.

Hence, the present study explains how the strength of the teacher and organizational settings can impact the setting of teaching. The Figure 1 illustrates the conceptual model of the study.

## 3 Materials and methods

### 3.1 Participants

The study looked at faculty members at Chinese universities to examine how teacher self-efficacy, well-being, emotional resilience, and enjoyment of teaching are related and what role institutional support and commitment to the profession play. Those actively involved in higher education teaching were chosen from the sampling pool by using a purposive method. It was an appropriate strategy for engaging people who have insight into the work environment in academia.

Faculty members were only allowed to respond to the survey after meeting the following requirements: they had to be teaching undergraduate or graduate courses in a Chinese university, must have at least one year of teaching experience, and must have given informed consent to respond to the survey. They had to be working in teaching capacity (i.e., not purely research or administration only) so they could legitimately answer questions on teaching pleasure, well-being, and resilience. Participants were not included when they were on long-term leave or secondment (and therefore not teaching), or when they had less than a year of teaching in their present institution (to avoid very new teachers without a settled teaching identity).

There were 273 valid responses collected, making it sufficient to run robust statistical analyzes, including structural equation modeling and mediation and moderation tests using regression. There was a variety among the sample participants in terms of gender, age, and their level of education, as shown in Table 1. A total of 54.2% of participants were males and 45.8% were females. Out of all respondents, 30.4% fell in the 25 to 35 age group, 46.5% were between the ages of 36 and 45, and 23.1% were 46 to 55 years old. This suggests that a majority of the teachers are experienced and are further along in their teaching careers. The majority of people (52.7%) who took part in the survey had master’s degrees or higher, whereas 14.7% had bachelor’s degrees and 32.6% had other forms of certification. This approach helps ensure the reliability of the data since it draws on expert opinions from multiple academic fields across China. Details can be seen in the Table 1.

**Table 1:** Demographical characteristics of the respondents.

Demographical variable	Category	*N*	Percentage
Gender	Male	148	54.2
	Female	125	45.8
Age	25–35 years	83	30.4
	36 to 45 years	127	46.5
	46 to 55 years	63	23.1
Education level	Bachelor’s degree	40	14.7
	Master’s degree or above	144	52.7
	Certification and others	89	32.6

### 3.2 Data collection procedure

Data was gathered using a questionnaire that was sent through an online survey. The survey was sent to academic mailing lists, university faculty networks, and professional teaching groups to make sure it was given to teachers across different parts of China. It took around 4 weeks to complete the data collection phase. The purpose of the study was briefly explained to participants, and their consent was obtained before inviting them to participate. They were further told their answers would be hard to match with their identities and that the researchers would ensure their confidentiality was protected during the research. Because the survey was online, respondents could contribute at a time that suited them, resulting in a large number of responses.

### 3.3 Measures

Self-efficacy, teacher well-being, emotional resilience, enjoyment in teaching, support from the school, and commitment to the profession were all assessed using a well-structured questionnaire and six validated scales. The responses for every construct were recorded on a five-point Likert scale, from 1 (Strongly Disagree) and going to 5 (Strongly Agree). Because of these tools, researchers and educators can study feelings and thoughts inside students that are relevant in university settings.

#### 3.3.1 Self-efficacy

The study used a scale of four items, which comes from Li et al. (2025), to evaluate how confident teachers feel about helping their students and handling classroom issues. Such a construct boosts both motivation and satisfaction among academics in their jobs.

#### 3.3.2 Teacher well-being

Limited to 7 questions, the survey applied a scale from Proietti Ergün and Dewaele (2021), to measure teacher feelings and concerns regarding their jobs. The scale measures various parts of the PERMA model (Positive Emotion, Engagement, Relationships, Meaning, Accomplishment) to show the overall well-being employees have at work.

#### 3.3.3 Emotional resilience

Researchers used 10 of the items from Campbell-Sills and Stein (2007) to measure emotional resilience, but adjusted the questions so they could be used in schools. This facet explains how a person resists adversity at work, deals with stress situations, and keeps up emotionally. Teachers’ enjoyment of teaching, well-being, and self-efficacy are all influenced through their acting as mediators. A careful protocol was used to adapt to achieve construct equivalence: items were contextualized in higher-education contexts, and terminology was aligned to classroom contexts.

#### 3.3.4 Teaching enjoyment

Teaching Enjoyment was rated on 7 items, adapted from measures created by Proietti Ergün and Dewaele (2021). The construct represents positive feelings people get from education, including feeling satisfied, interested, and involved in learning. The main aim of this study is to focus on this outcome.

#### 3.3.5 Institutional support and professional commitment

The assessment of Institutional Support included 4 statements from Chang and Choi (2007), reflecting perceptions of how well employees are cared for by the organization. Professional Commitment was measured with 5 items that came from Abdulaziz et al. (2022). To find out if the effect of emotional resilience on teaching enjoyment changes with the level of support and commitment available, researchers examined these constructs as moderators.

### 3.4 Data analysis

The collected data from the 273 university faculty members were analyzed using IBM SPSS (version 26) and AMOS (version 24) software. To explore the relationships among the study variables and to test the hypothesized effects of mediation and moderation, researchers applied a mix of descriptive, correlational, and inferential statistical methods. First, descriptive statistics were computed to summarize the main features of the participants and the average and standard deviations of central variables. Next, Pearson correlation analysis was performed to determine the relationship and direction between self-efficacy, well-being, emotional resilience, teaching enjoyment, institutional support, and professional commitment. The findings gave the first insight into how mediating and moderating processes play a part in the model.

SPSS was used to conduct a multiple regression analysis in order to assess how much self-efficacy and teacher well-being explained the degree to which teachers found their job rewarding. In AMOS, the researchers checked to see if emotional resilience acts as a mediator between self-efficacy, well-being, and enjoyment in teaching. To decide if the indirect effects were important, the researchers calculated 95% confidence intervals (Wang et al., 2022). Besides, it was examined through hierarchical regression in SPSS whether feeling committed to their profession and receiving institutional support soften the link between emotional resilience and teacher enjoyment. Such interaction terms were used in the models to check for different effects depending on conditions. In the end, model fit was examined using Confirmatory Factor Analysis in AMOS and relevant indices (CFI, TLI, IFI, RMSEA, and SRMR) to check if the measurement model was suitable for performing hypothesis testing in SEM (Byrne, 2013).

### 3.5 Reliability and validity testing

The strength and accuracy of the data are tested here by reliability and validity testing.

Both reliability and validity checks were performed to ensure that the instruments were robust. To check internal consistency reliability, Cronbach’s alpha, Composite Reliability (CR), and Average Variance Extracted (AVE) were used for every construct. Table 2 reveals that all constructs had very good reliability, as Cronbach’s alpha values surpassed the minimum recommended value of 0.70 (Bernardi, 1994). All of emotional resilience (*α* = 0.929), teacher well-being (*α* = 0.913), teaching enjoyment (*α* = 0.927), institutional support (*α* = 0.920), professional commitment (*α* = 0.854), and self-efficacy (*α* = 0.890) have excellent internal consistency. Each composite reliability for the scales was above 0.70, which further proves their reliability. For all measures, AVE was greater than 0.5, which indicates that convergent validity is appropriate. These findings prove that the scales accurately measure the desired psychological and organizational aspects among Chinese university faculty members. Details can be seen in the Table 2.

**Table 2:** Result of scale’s reliability test.

Variable	Number of items	Cronbach alpha	AVE	CR
Emotional Resilience	10	0.92	0.57	0.93
Teachers Well-being	7	0.91	0.60	0.91
Teaching Enjoyment	7	0.92	0.64	0.92
Institutional Support	4	0.92	0.74	0.92
Professional Commitment	5	0.85	0.54	0.85
Self-Efficacy	4	0.89	0.67	0.89

Construct validity was assessed through convergent validity. Convergent validity was confirmed by examining the Average Variance Extracted (AVE) for each construct. Table 2 shows that AVE values exceeded the recommended threshold of 0.50 (Cheung et al., 2024). It showed that each latent variable adequately explains the variance of its items. Additionally, Composite Reliability (CR) values for all constructs were above 0.70 (Sujati and Akhyar, 2020) which showed a strong internal consistency (see Table 2). In Table 3, the model showed a good fit, with χ^2^/df = 1.55 and CFI = 0.95, TLI = 0.94, IFI = 0.95, and SRMR = 0.04, all of which are within or above the usual recommendations (CFI, TLI, IFI > 0.90; SRMR < 0.08) (Sathyanarayana and Mohanasundaram, 2024). An RMSEA of 0.04 demonstrates once again that the model is fitted well to the data, as it is lower than the preferred cut-off of 0.08. All these indicators confirm that the model measures education from several perspectives and distinguishes different latent constructs. Therefore, the results of this validation study indicate the psychometric soundness of the questionnaire, making it suitable for mediation and moderation analysis. Details can be seen in the Table 3.

**Table 3:** Validation of factor analysis.

Fit index	χ^2^/df	CFI	TLI	NFI	IFI	SRMR	RMSEA
Reference value	<5.00	>0.90	>0.90	>0.90	>0.90	<0.08	<0.08
Test value	1.55	0.95	0.94	0.87	0.95	0.04	0.04

The discriminant validity results using the HTMT criterion show the relationships between the study constructs: ERS, TWLB, TE, IS, PC, and SEF (Ab Hamid et al., 2017). The HTMT values between ERS, TWLB, TE, IS, PC, and SEF resulted from 0.01 to 0.81. However, values below 0.85 indicate adequate discriminant validity. It depicts that the constructs are empirically distinct. In this regard, most HTMT values were below the threshold, which supported discriminant validity. However, there were also higher values, such as 0.81 between ERS and TE, and 0.77 between TWLB and SEF. It indicated moderately strong associations, which suggested that these constructs are related but still distinguishable. The low values between IS, PC, and other constructs ranged from 0.01 to 0.24, clearly differentiating from the other variables. Thus, the HTMT results indicate that the constructs in the model maintain sufficient discriminant validity (Afthanorhan et al., 2021). In this regard, each construct captures a unique aspect of the theoretical framework and reflects expected conceptual relationships (Table 4).

**Table 4:** Discriminant validity HTMT.

Variables	ERS	TWLB	TE	IS	PC	SEF
ERS	-					
TWLB	0.61	-				
TE	0.81	0.70	-			
IS	0.16	0.01	0.24	-		
PC	0.12	0.19	0.06	0.15	-	
SEF	0.67	0.77	0.72	0.04	0.17	-

## 4 Results

### 4.1 Descriptive statistics and correlations

Descriptive statistics and Pearson correlation coefficients were computed to explore the distribution and interrelationships among the study variables. As shown in Table 5, the mean scores for the key variables ranged between 3.20 and 3.57, indicating a moderate to high level of agreement among respondents on constructs such as self-efficacy (*M* = 3.58, SD = 1.16), teacher well-being (*M* = 3.52, SD = 1.10), emotional resilience (*M* = 3.27, SD = 0.95), and teaching enjoyment (*M* = 3.31, SD = 1.04).

**Table 5:** Descriptive statistics and the interconnection between the variables.

Variable	*M*	SD	1	2	3	4	5	6	7	8	9
1. Gender	1.46	0.49	-								
2. Age	1.93	0.72	0.03	-							
3. Education	2.18	0.66	−0.00	0.20^**^	-						
4. PC	2.70	1.00	0.03	0.08	0.20^**^	-					
5. TWLB	3.52	1.09	0.01	−0.02	−0.05	−0.17^**^	-				
6. ERS	3.27	0.95	0.05	0.04	−0.02	−0.11	0.57^**^	-			
7. TE	3.30	1.04	0.03	0.06	0.02	−0.05	0.64^**^	0.75^**^	-		
8. SE	3.57	1.15	0.05	0.05	−0.00	−0.15^*^	0.69^**^	0.61^**^	0.65^**^	-	
9. IS	3.20	1.30	−0.05	0.13^*^	0.16^**^	0.13^*^	0.01	0.15^*^	0.22^**^	0.04	-

PC, professional commitment; TWLB, teacher well-being; ERS, emotional resilience; TE, teaching enjoyment; SE, self-efficacy; IS, institutional support.

*Refers to 0.1 and **Refers to 0.05 levels of significance.

Correlation analysis revealed several statistically significant positive associations. Teaching enjoyment was strongly correlated with emotional resilience (*r* = 0.75, *p* < 0.01), teacher well-being (*r* = 0.64, *p* < 0.01), and self-efficacy (*r* = 0.65, *p* < 0.01), indicating that higher internal resources were associated with greater teaching enjoyment. Additionally, emotional resilience was significantly linked to both well-being (*r* = 0.57) and self-efficacy (*r* = 0.61), supporting its potential role as a mediator in the model. Details can be seen in the Table 5.

### 4.2 Regression analysis

A multiple regression analysis was conducted to examine the influence of demographic variables and internal resources on teaching enjoyment. As presented in Table 6, the overall model was statistically significant (*R* = 0.71, *R*^2^ = 0.50, *F* = 54.46, *p* < 0.00), indicating that approximately 50.5% of the variance in teaching enjoyment was explained by the predictors.

**Table 6:** Results of the intermediary regression model analysis.

Outcome variable	Predictor variable	*R*	*R*^2^	*F*	*Β*	*T*
TE	Gender	0.71	0.50	54.461***	0.00	0.11
	Age				0.04	0.97
	Education				0.03	0.79
	TWLB				0.37	6.22***
	SE				0.39	6.51***

TE, teaching enjoyment; TWLB, teacher well-being; SE, self-efficacy.

***Refers to *p* < 0.01, it is the significance level.

Among the demographic variables (gender, age, and education) none showed a statistically significant effect on teaching enjoyment. However, both teacher well-being (*β* = 0.37, *t* = 6.22, *p* < 0.00) and self-efficacy (*β* = 0.39, *t* = 6.51, *p* < 0.00) were significant positive predictors. These results suggest that faculty members who report higher levels of psychological well-being and self-belief in their teaching capabilities are more likely to enjoy their teaching roles. The findings reinforce the importance of internal psychological resources as key drivers of positive teaching experiences in higher education settings. Details can be seen in the following Table 6.

### 4.3 Mediation analysis

To examine whether emotional resilience mediates the relationship between internal resources and teaching enjoyment, a bootstrapped mediation analysis was conducted using 5,000 resamples and 95% confidence intervals. The analysis tested two indirect paths: (Kamboj and Garg, 2021) teacher well-being (TWLB), emotional resilience (ERS), teaching enjoyment (TE) and (Thumvichit, 2024) self-efficacy (SE), ERS and TE.

As shown in Table 7, both mediation pathways were statistically significant. The total indirect effect of teacher well-being on teaching enjoyment through emotional resilience was 0.3238 (Boot SE = 0.02; 95% CI: 0.26 to 0.38), with the confidence interval not containing zero, indicating significant mediation. Similarly, the indirect effect of self-efficacy on teaching enjoyment via emotional resilience was 0.30 (Boot SE = 0.03; 95% CI: 0.24 to 0.37), also confirming significant mediation.

**Table 7:** Mediation effect test results.

Model effect	Effect size	Boot SE	95%CI		Effect proportion
			LLCI	ULCI	
Total effect: TWLB → ERS	0.61	0.04	0.52	0.70	
Total effect: SE → ERS	0.59	0.04	0.51	0.67	
Direct effect: SE → ERS	0.28	0.04	0.19	0.36	47.6%
Direct effect: TWLB → ERS	0.30	0.04	0.22	0.38	49.8%
Total indirect effect					
SE → ERS → TE	0.30	0.03	0.24	0.37	52.2%
TWLB →ERS → TE	0.32	0.02	0.26	0.38	52.7%

TWLB, teacher well-being; SE, self-efficacy; ERS, emotional resilience; TE, teaching enjoyment.

The direct effects of TWLB and SE on ERS were both strong and significant, with effect sizes of 0.30 and 0.28, respectively, further supporting emotional resilience as a key psychological bridge. These results highlight the critical role of emotional resilience in transforming internal psychological strengths (well-being and self-efficacy) into enhanced teaching enjoyment, confirming its mediating function within the model. Details can be seen in the following Table 7.

### 4.4 Moderation analysis

Moderation analysis was performed using hierarchical regression to test whether professional commitment (PC) and institutional support (IS) moderate the relationship between emotional resilience (ERS) and teaching enjoyment (TE). Interaction terms (ERS × PC and ERS × IS) were computed and entered into the regression model in the final step.

As shown in Table 8, the interaction between emotional resilience and professional commitment was statistically significant (*β* = 0.09, *t* = 2.42, *p* < 0.01), suggesting that professional commitment strengthens the positive effect of emotional resilience on teaching enjoyment. In contrast, the interaction between emotional resilience and institutional support was not statistically significant (*β* = 0.06, *t* = 1.51, *p* > 0.05), indicating that institutional support did not significantly alter this relationship in the sample. Overall, the findings highlight professional commitment as a meaningful contextual factor that enhances the influence of personal resilience on teaching enjoyment, whereas institutional support may play a less direct moderating role in this dynamic.

**Table 8:** Moderation effect test results with hierarchical regression.

Outcome variable	Predictor variable	*R*	*R*^2^	*F*	*β*	*t*
TE	ERS*PC	0.75	0.57	121.66***	0.0.097	2.42**
	ERS*IS	0.76	0.58	124.57***	0.061	1.51

TE, teaching enjoyment; ERS, emotional resilience; PC, professional commitment; IS, institutional support.

*Refers to 0.1 and **Refers to 0.05 levels of significance.

In conclusion, the data analysis has supported the proposed hypotheses to a large extent. *H1* and *H2* were supported because both teacher self-efficacy and teacher well-being had significant positive effects on teaching enjoyment. *H3* and *H4* were also supported because emotional resilience significantly mediated the relationships between self-efficacy and teaching enjoyment, and between teacher well-being and teaching enjoyment. It has been indicated by the significant indirect effects in the bootstrapped mediation analysis. Regarding the moderating hypotheses, *H6* was also supported because professional commitment strengthened the positive effect of emotional resilience on teaching enjoyment. However, *H5* was not supported because institutional support did not significantly moderate this relationship. Thus, these results confirm that internal psychological resources play a central role in teaching enjoyment. In this regard, professional commitment significantly improves the impact of emotional resilience.

## 5 Discussion of findings

Figure 1 has been revisited to support the interpretation of the confirmed pathways. It clarifies how the hypothesized relationships among self-efficacy, well-being, emotional resilience, and teaching enjoyment emerged in the results.

### 5.1 Teacher self-efficacy and teaching enjoyment in the higher education faculty

The results showed a significant positive relationship between teacher self-efficacy and teaching enjoyment. Even after accounting for potential mediating variables, the positive effect of self-efficacy on teaching enjoyment remained significant, indicating that as the level of teacher self-efficacy increased, the level of teaching enjoyment also significantly rose, thus supporting Hypothesis 1. This finding is consistent with prior research. For example, Schunk and DiBenedetto (2021) found that self-efficacy significantly enhances teachers’ intrinsic motivation, which in turn effectively promotes their engagement and enjoyment in teaching. Teacher self-efficacy influences teaching enjoyment through multiple mechanisms. First, positive feedback and confidence in their abilities can enhance teachers’ perceptions of their own competence, which is central to Bandura’s (1977) self-efficacy theory. When teachers receive positive reinforcement, their self-confidence increases. This, as a result, further stimulates their enjoyment in the classroom. Additionally, when teachers are confident in their abilities, they are more likely to use student-centered approaches, stay committed through hard times, and feel more satisfied in their jobs. This finding underscores that the role of teachers extends beyond just delivering content, they should also focus on strengthening their self-belief, as it has a profound impact on their sense of engagement and overall enjoyment in teaching.

Based on SE theory, this can be elucidated as follows: the faculty will more likely feel a sense of clear mastery when they believe they can effectively meet the instructional demands and have the ability to influence student outcomes, and thereby derive more pleasure in teaching. Teacher enjoyment and self-efficacy in higher education are positively related. This lends credence to the notion that self-efficacy is actually a psychological resource upon which teachers rely to become more engaged and feel more pleasure in the teaching process (Xiao et al., 2022; Bing and Cai, 2025).

### 5.2 Teacher well-being and teaching enjoyment in the higher education faculty

The results showed a significant positive relationship between teacher well-being and teaching enjoyment, thus supporting Hypothesis 2. This finding aligns with prior research. For example, Van Dierendonck and Lam (2023) emphasized that psychological well-being includes self-acceptance, positive relations with others, autonomy, environmental mastery, personal growth, and purpose in life. Teachers who experience greater well-being are more likely to feel a sense of purpose, autonomy, and recognition in their work, leading to increased enjoyment of teaching (Hammoudi Halat et al., 2023). Teacher wellness helps improve their satisfaction with teaching in several ways. If teachers feel good and content, they are more ready to manage the challenges in their job, which leads to greater enjoyment and satisfaction as teachers. Self-Determination Theory points out that having autonomy at work and good relationships with colleagues lead to better psychological well-being, which supports more enjoyment in teaching. It proves that institutions can help faculty feel better by providing support, which leads to less stress, greater satisfaction at work, and better teaching. When faculty well-being is valued, teachers are more likely to be engaged and motivated, which results in higher job satisfaction and happiness in their roles.

### 5.3 Emotional resilience as a mediator between teacher self-efficacy and teaching enjoyment

It was discovered that emotional resilience both directly and positively contributed to teachers’ enjoyment of their work and also played an indirect part by influencing teaching enjoyment through its mediation of the relationship between teacher self-efficacy and teaching enjoyment, thus confirming Hypothesis 3. The result is in accordance with the theoretical framework that teachers need emotional resilience to handle the problems they experience in higher education. According to Sisto et al. (2019), being able to endure stress and hard times without trouble to mental and emotional well-being is part of being emotionally resilient. Being emotionally resilient as a teacher allows them to deal with changes or pressures from work and make teaching more satisfying. Being resilient is important for turning self-efficacy and well-being into activities like greater engagement and enjoyment in teaching (Bagdžiūnienė et al., 2023). Similarly, Taylor (2013) points out that by being resilient, teachers can use their own strengths and also the resources available to them to overcome obstacles and stay dedicated to their work. Teachers who are more resilient manage challenges well, make the most of feedback, and are dedicated, which helps them enjoy teaching for a longer period. The study shows that having emotional resilience is important for connecting how much a teacher feels effective with their degree of enjoyment in teaching.

Revolving around the concept of self-efficacy theory, the result suggests that teachers who believe in their teaching competencies contribute to emotional strength that acts as a factor to continued and intensified enjoyment in educational practice. Stress-resistant teachers can overcome frustrations and convert confidence into a sustained positive classroom life. This tendency correlates with previous research that links self-efficacy to resilience and well-being, and indicates that resilience is a direct predictor of teaching enjoyment and mediates the relationships between efficacy, well-being, and positive professional outcomes. In such a way, the results support the idea that reinforcing self-efficacy fosters resilience, which is a fundamental psychological route to increased enjoyment in teaching (Wang et al., 2024a; Bagdžiūnienė et al., 2023; Xiao et al., 2022).

### 5.4 Emotional resilience as a mediator between teacher well-being and teaching enjoyment

The findings showed that emotional resilience significantly mediates the relationship between teacher well-being and teaching enjoyment, which supported Hypothesis 4. This result agrees with earlier studies, which show that emotional resilience helps teachers transform their well-being into more satisfaction and enjoyment in teaching. For instance, Kun and Gadanecz (2022) pointed out that having strong well-being gives teachers emotional strength and the ability to stay joyful in their profession, which is needed for good teaching. Being resilient helps teachers keep a positive attitude and remain emotionally steady in tough times, which improves their happiness and enjoyment at work. Those who feel content and positive in their jobs usually manage stress well, cope with changes, and experience less burnout (Nurfatihah Razali and Sukor, 2023). Having resilience allows teachers to turn their emotional and psychological stability into satisfaction with their work, no matter how stressful or challenging the situation. This study points out that caring for teachers’ well-being in colleges and universities helps teachers feel better emotionally and enjoy their jobs more. It is important for institutions to help teachers by providing resources and making sure the environment is supportive, which leads to better emotional and professional outcomes for teachers. Using this approach will ensure teachers have long-term job satisfaction and ultimately contribute to a more engaged and effective teaching workforce.

In the SDT context, the observation that emotional resilience mediates the relationship between teacher well-being and teaching enjoyment can be interpreted as follows: when educators have higher levels of well-being (characterized by autonomy, connectedness, and purpose), they become more resilient, thereby being able to retain positive experiences in teaching and fun in their jobs. The mediation effect highlights that well-being is needed, but is not enough. Resilience acts as the mental process that converts well-being into long-term pleasure (Wang et al., 2024a; Zhang, 2023).

### 5.5 Institutional support as a moderator between emotional resilience and teaching enjoyment

The findings demonstrated that institutional support does not significantly moderate the relationship between emotional resilience and teaching enjoyment, which means Hypothesis 5 is not supported. According to this finding, emotional resilience helps teachers enjoy their work, but the role of institutional support in making this effect stronger was not significant in the current study. On one level, the result does not align with expectations that institutional job-resources amplify the effect of personal resources such as resilience, yet resilience itself overshadows structural support in predicting positive outcomes (e.g., structural resources were weaker predictors than individual resilience) (Reintjes et al., 2025). Some possible explanations underlie the non-significant moderation. Perhaps the institutional support construct lacked adequate variance (e.g., uniformly high support across respondents), limiting its capacity to interact meaningfully with resilience. Besides, in a higher-education faculty setting, support may be delivered uniformly, minimally, or perceived differently, so the moderating effect may vanish. Furthermore, in some academic cultures, faculty rely more heavily on internal factors (resilience, autonomy) than external institutional supports, therefore dampening the moderation effect.

The absence of moderation does not mean institutional support is unimportant; rather, it suggests that the pathway from resilience to teaching enjoyment operates fairly independently of institutional support in this context, or that the support provided is not sufficiently tailored to bolster that specific link. Future research should employ more refined measures of support (e.g., mentoring, peer-networks, leadership responsiveness), examine interaction effects under varying levels of institutional resourcefulness, and explore cultural moderators to unpack why support sometimes does not strengthen resilience.

According to research, support from institutions can improve how faculty feel and motivate them to teach (Zhao, 2024). Getting support from the institution, for example, with resources, mentoring, and training, can make teachers feel less stressed and more resilient, which may improve their enjoyment of teaching. Still, even with these known benefits, the current research finds that institutional help might not always significantly influence the direct relationship between emotional resilience and teaching enjoyment. Different factors might explain why there is not a noticeable moderating effect, such as the specific context of the institution, the type of support offered, or the individual differences among faculty members in how they perceive and utilize institutional support. This research hints that although general support is important for overall well-being, the effects of such support on teachers’ enjoyment of teaching through emotional strength might need further attention, especially with respect to the type and extent of support provided.

### 5.6 Professional commitment as a moderator between emotional resilience and teaching enjoyment

Results indicated that professional commitment significantly moderates the relationship between emotional resilience and teaching enjoyment, which supports Hypothesis 6. The study’s finding matches what is found in previous studies, which stress that a strong commitment to the profession boosts teachers’ resilience and satisfaction with their work. Those who are enthusiastic about their jobs tend to spend more effort improving teaching, try out new reflective practices, and stay active in their roles despite any pressures from the institution (Begum, 2022). Being dedicated to their profession means educators can manage the difficulties of teaching, and this results in more happiness in their teaching. Based on Shao’s (2023) findings, passionate and committed teachers persist in their work, are happier in their roles, and tend to stay involved despite facing various challenges at work. They consider teaching problems as chances to develop, which makes them more emotionally strong and improves their teaching experience. When teachers feel strongly connected to their profession, they usually remain motivated, manage difficulties, and keep improving as teachers, which results in sustained job satisfaction and enjoyment. It emphasizes that being professionally committed to your profession is important for strengthening the relationship between emotional resilience and teaching enjoyment.

The finding based on the SDT is that highly committed faculty members have a better opportunity of shifting their resilience to pleasure in their teaching work. A strong association exists between an increased professional commitment and teacher engagement and perseverance. In other words, committed teachers direct their feelings of resilience to a greater extent and therefore love teaching. It demonstrates that professional commitment also enhances the resilience-enjoyment relationship at the contextual level (Dong and Xu, 2022; Mangarin and Macalde, 2024).

The unique socio-cultural and institutional context of Chinese higher education likely influenced the study’s results in important ways. In China’s considerably hierarchical organizations, with comparatively high power-distance, faculty experience stronger top-down mandates, less autonomy, and fewer opportunities for open feedback or student-centered negotiation, which can diminish the translation of self-efficacy into enjoyment under normal forms of institutional support (Wang and Fränti, 2022; Qian and Liu, 2021). Collectivist values and group-oriented work cultures mean that professional commitment carries greater weight in motivating faculty, boosting the effect of resilience on enjoyment when commitment is high. At the same time, extensive performance-based pressures in Chinese universities, driven by national programs and talent schemes, place significant demands on teaching staff and shape their well-being and resilience pathways (Zheng and Postiglione, 2024; Hu, 2024). Moreover, institutional supports can be routinely offered but standardized across contexts rather than individually tailored, which could explain the non-significant moderating effect of institutional support in this setting. The overarching administrative culture thus frames how self-efficacy, well-being, resilience, and enjoyment are experienced and manifested among Chinese faculty, meaning the observed paths reflect context-specific dynamics of teaching in China rather than universal patterns.

## 6 Research implications

As a result of this study, there are now several new insights into the psychology of teachers in higher education. It adds to the research on positive psychology since it shows that self-efficacy and well-being, which are needed personal qualities, both go well with the enjoyment teachers experience in their role. This study confirms that self-efficacy theory and conservation of resources theory apply, as people with strong abilities tend to do well emotionally at their workplace (Proietti Ergün and Dewaele, 2021). In addition, emotional resilience is discussed as a critical psychological factor that uses personal resources to create useful emotional experiences (Campbell-Sills and Stein, 2007).

In addition, this work adds to the literature by mapping out a subtle link between teacher self-efficacy and well-being and teaching enjoyment via the important mediator of emotional resilience. While prior research has identified self-efficacy’s link to engagement and resilience’s connection to well-being (for example, self-efficacy and resilience were shown to predict teacher burnout) (Wang et al., 2024a; Li, 2023; Heng and Chu, 2023), this study moves beyond by demonstrating how resilience mediates the effect of both self-efficacy and well-being on enjoyment in a higher-education faculty context, and further identifies professional commitment as a moderator of the resilience-enjoyment link. This model enriches existing educational psychology frameworks by showing not just what matters (efficacy, well-being, resilience) but how and when those personal resources translate into enjoyment of teaching.

This study yields concrete practical contributions. As self-efficacy strongly predicted teaching enjoyment, universities should implement structured mentoring, peer-coaching, and faculty-development pathways (formal observation, formative feedback, certified mentor training) to build instructional confidence; approaches shown to strengthen teaching skills and efficacy in higher-education contexts (Rahman, 2023; Le et al., 2024). Given that emotional resilience mediated both the self-efficacy-enjoyment and well-being-enjoyment links, institutions should fund short, focused resilience programs (skills workshops, cognitive behavioral therapy-informed coping modules, reflective practice groups) that directly target emotion regulation and adaptive coping (Hollaar et al., 2025). As professional commitment amplified resilience’s payoff for enjoyment, create recognized teaching career tracks, teaching fellowships, and communities of practice that valorize pedagogy and strengthen professional identity (Halal Orfali et al., 2024). These findings also align with global sustainability priorities. Thus, the present study directly contributes to SDG 3: Good Health and Well-Being. It focuses on promoting psychological health in professional settings. Furthermore, the enhancement of teaching enjoyment and professional commitment also supports SDG 4: Quality Education. This is because emotionally stable and motivated educators holds immense importance for improving learning environments and educational quality.

The non-significant moderating effect of generic institutional support suggests moving away from one-size-fits-all welfare toward targeted supports (teaching-load relief for course redesign, small instructional grants, rapid peer support) that align with faculty needs (Wang, 2024). Furthermore, embed continuous evaluation (pre/post measures, program audits, usage metrics) to identify which interventions convert faculty resources into sustained enjoyment and to reallocate funding to high-impact initiatives (Cotta et al., 2024). These implications offer universities practicable routes to boost faculty wellbeing, resilience, and enduring teaching enjoyment.

## 7 Conclusion

### 7.1 Summarizing the research findings

The primary aim of this study was to investigate how teacher self-efficacy and well-being contribute to teaching enjoyment, with a particular focus on the mediating role of emotional resilience and the moderating effects of professional commitment and institutional support. The results provide robust support for the hypothesized model. Being confident and motivated as teachers, as well as being well supported by colleagues, led to teachers reporting more enjoyment, suggesting that inner strength and support from others matter for a fulfilling work environment in schools. Evidently, emotional resilience played a major role in this process by changing people’s inner strengths into lasting positive emotions on the job. About moderation, having a strong professional commitment enhanced the positive connection between higher emotional resilience and greater joy in teaching, but institutional support did not play a significant role. This indicates that specific capabilities of individual teachers play a major role in making their work in education successful and long-lasting.

### 7.2 Identifying research limitations

While this study provides valuable insights into the psychological mechanisms underlying teaching enjoyment, several limitations must be acknowledged. First, the cross-sectional design restricts the ability to draw causal inferences. Although mediation and moderation analyses offer theoretical pathways, longitudinal data would be necessary to confirm the temporal order of the relationships. Second, all variables were measured through self-reported surveys, which may be susceptible to social desirability bias and common method variance. Incorporating multi-source data, such as peer evaluations or institutional performance records which could enhance objectivity. Third, the study’s sample was limited to university faculty in China, which may constrain the generalizability of findings to other cultural, institutional, or educational settings. Additionally, the non-significant moderating effect of institutional support might be context-specific, possibly reflecting variations in how support is perceived or provided in Chinese academic environments. The study’s limitations should be considered when looking at its main findings and what they mean. The mediation frameworks assume temporal precedence even though the variables were evaluated at one time, limiting the ability to establish the order of mediation. Indirect effects may be significantly biased when obtained through cross-sectional mediation analyses (Georgeson et al., 2023). Furthermore, the use of self-report data creates potential bias. Therefore, these limitations imply that, although it is possible to draw associations, causal conclusions should be assessed with care; longitudinal studies are required to support the hypothesized mechanisms in the future.

### 7.3 Suggesting future research directions

Based on the current results, future study can examine different aspects to flatten the wellbeing–resilience–enjoyment chain in higher education. With longitudinal designs, researchers could study more thoroughly why and how academic work engagement is associated with outcomes and changes. Also, blending different ways of gathering information. It includes interviews, advice from colleagues and institution’s statistics which can help understand teachers’ internal lives and reduce the chances of reporting bias.

It would also help to analyze these relationships in different societies and academic systems, in order to see if the findings from China agree with those in other parts of the world. It is important to further study that why institutional support did not play much of a role in moderating. Additional studies might look into how assistance in specific areas (for example, emotional, administrative, developmental) affects emotional resilience differently. Furthermore, zooming into job satisfaction, heavy workload and signs of burnout could reveal extra details about the relationship between resilience and enjoying teaching (Li et al., 2025). More studies are now needed to see how teaching in digital or hybrid environments affects the mental and physical well-being of educators (Haleem et al., 2022).

## Data Availability

The raw data supporting the conclusions of this article will be made available by the authors, without undue reservation.
